# Combined effect of microbial and non-microbial biostimulants on wheat growth, yield, and grain nutritional quality under sandy soil conditions

**DOI:** 10.1186/s12870-026-09659-4

**Published:** 2026-08-13

**Authors:** MMS Abdallah, TNM El Sebai, BA Bakry, MS Sadak, H M S El-Bassiouny

**Affiliations:** 1 Botany Department, Agricultural and Biological Research Institute, National Research Centre, 33 El Bohouth Street, P.O. 12622. Dokki, Giza, Egypt; 2 Agricultural Microbiology Department, Agricultural and Biological Research Institute, National Research Centre, 33 El Bohouth Street P.O. 12622. Dokki, Giza, Egypt; 3 Field Crops Research Department, Agricultural and Biological Research Institute, National Research Centre, 33 El Bohouth Street P.O. 12622. Dokki, Giza, Egypt

**Keywords:** Banana peel, Biofertilizer, Growth, Nutritional quality, Tryptophan, Wheat

## Abstract

**Background:**

The agricultural use of sandy soils has expanded worldwide to meet the increasing demand for food and other agricultural commodities resulting from rapid population growth and urbanization. However, the inherent limitations of these soils, particularly their low capacity to retain water and nutrients, pose major challenges to sustainable crop production. Accordingly, field experiments were conducted to assess the effectiveness of integrated microbial and non-microbial biostimulants in improving the performance of wheat (*Triticum aestivum* L. cv. Sakha-94). Wheat grains were co-inoculated with *Trichoderma harzianum* and *Aspergillus niger* as phosphate-solubilizing fungi (TD + PSF), and plants were treated with potassium sulfate (K_2_SO_4_ 200 mg/l), tryptophan (Try 50 mgL-^1^), K_2_SO_4_ (200 mgL-^1^) + Try (50 mgL^−1^), and two concentrations of banana peel extract (BPE) (500 and 1000 mgL-^1^).

**Results:**

Yield performance was substantially improved, with increases of 34.2%, 38.9%, and 44.1% in straw yield, biological yield, and grain yield, respectively. Supporting these yield gains, growth characteristics and physiological attributes were significantly enhanced. The combined application of TD + PSF and BPE1000 produced the highest increases in chlorophyll a (31.4%), chlorophyll b (83.6%), total pigments (48.2%), and phenolic compounds (18.9%). Indole-3-acetic acid (IAA) content increased significantly following co-inoculation, with the greatest enhancement (34.9%) recorded under the K₂SO₄ + tryptophan treatment combined with TD + PSF. Grain nutritional quality was also enhanced. The BPE at 1000 mg L^−1^ combined with TD + PSF treatment resulted in the highest carbohydrate content (12.5%), whereas BPE500 combined with TD + PSF achieved the greatest increase in grain protein content (33.2%). The combination of K₂SO₄ + tryptophan and TD + PSF significantly improved mineral accumulation, increasing N, P, K, and Mg contents by 33.2, 41.7, 16.2, and 44.2%, respectively. In addition, the highest water productivity (44.12%) was obtained with BPE1000 combined with TD + PSF. The ratio of essential to non-essential amino acids in the grain yield was enriched by approximately 12–15% in plants treated with the combination of TD + PSF and other treatments. This enrichment indicates an improvement in the nutritional quality of the wheat grains, enhancing their value for human dietary needs.

**Conclusion:**

Among the tested treatments, the combined application of (TD + PSF) and banana peel extract (1000 mg L⁻^1^) produced the greatest improvements in wheat growth, yield, and grain quality. These findings demonstrate that banana peel extract, particularly when combined with beneficial fungi, is a promising, low-cost, and eco-friendly biostimulant for wheat cultivation in reclaimed sandy soils, while promoting the sustainable utilization of agricultural waste.

**Supplementary Information:**

The online version contains supplementary material available at 10.1186/s12870-026-09659-4.

## Introduction

Wheat (*Triticum aestivum* L.) is a strategic and vital crop for international food security as it is an essential source of calories and protein in more than 80 countries [1]. The demand for wheat is projected to rise as the global population increases, with the world’s population projected to range from ≃ 10 billion in 2050 [2]. In Egypt, there is an immense gap between the output and consumption of wheat (˃50%) [3]. Hence, to reduce this gap, Egypt has tried to increase wheat production vertically by developing new varieties through conventional and modern breeding and horizontally through the expansion of desert land reclamation called “reclaimed sandy soil”.

Biostimulants are known to enhance plant performance under both normal and stress conditions. In this study, we focus on reclaimed sandy soil, which is characterized by its coarse texture, low water-holding capacity, poor nutrient retention, and susceptibility to drought stress. These properties create challenging abiotic stress conditions for crop growth, including limited moisture availability and nutrient deficiencies. Therefore, evaluating biostimulant effectiveness under these conditions is crucial to developing sustainable strategies for improving crop productivity in such marginal soils. According to Rai et al. [4], biostimulants have recently been regarded as a potentially innovative method to enhance crop productivity and plant growth under normal and stress conditions. This represents a significant scientific breakthrough in ensuring that prospective food production is sustainable. Therefore, it is crucial to develop sustainable methods to mitigate the adverse effects of abiotic stress on plant performance. Using environmentally friendly substances, similar microbial and non-microbial biostimulants, is one of the most promising approaches to lessen the detrimental influences of abiotic stressors on plants [5]. When applied to plants, biostimulants, organic or inorganic substances, and/or microorganisms stimulate many processes that promote growth and production [6] and resilience to stress. Biostimulants come in a variety of forms and may be categorized into six main groups according to the source of the raw material: seaweed, plant extracts, protein hydrolysates, humic substances, inorganic chemicals, and microorganisms [7].

The natural phosphorus cycle depends heavily on microorganisms, and using phosphate-solubilizing microorganisms (PSMs) has been proposed as an inexpensive way to increase the agronomic efficiency of insoluble phosphates. To decrease phosphorus shortages and protect plant productivity, a great amount of expensive chemical phosphate fertilizers is utilized worldwide every year. Microorganisms such as bacteria, fungi, and actinomycetes can promote phosphorus solubilization and improve crop yields [8]. Through exchange processes, chelation, and acidity, PSMs transform insoluble phosphate into a soluble form [9]. Amongst the fungal genera with phosphate solubilization capability are *Aspergillus, Penicillium, Trichoderma, Eupenicillium,* and *Gliocladium* [10]. *Aspergillus niger* is a versatile fungus that plays a role in the solubilization of potassium and phosphorus [11], as well as in promoting the biosynthesis of phytohormones [12, 13]. The use of *A. niger* has led to enhancements in soil quality, the microbial community, and lettuce yields in barrier soil [14]. *A. niger* is capable of solubilizing various elements such as phosphorus, potassium, calcium, and other minerals found in rocks, making them available in a form that plants can easily absorb. Furthermore, *A. niger* has been shown to produce plant growth-promoting and protective substances, including sesquiterpenes (β-caryophyllene), indole-3-acetic acid (IAA), gibberellins, as well as 2-carboxymethyl-3-hexyl-maleic anhydride and 2-methylene-3-hexyl-butanedioic acid [13].

The solubilization ability of PSF relies on the insoluble inorganic phosphate exporter, the types of carbon, nitrogen, and metal ions in the soil, in addition to culture conditions [15, 16]. Akbari et al. [17] stated that *Trichoderma afroharzianum* T22 could reinforce *Solanum lycopersicum* seed germination under various stressors by mitigating oxidative damage in osmotically challenged conditions.

Potassium (K) is an essential macronutrient and follows nitrogen and phosphorus in terms of the importance of plant nutrition. It performs vital functions in various processes, including cation–anion balance, stomatal movement, energy transfer, protein synthesis, osmoregulation, photosynthesis, and enzyme activation [18]. Potassium is needed for plant growth and development, particularly in soils that suffer from poor fertility and a deficit in nutrient elements.

L-tryptophan is an essential amino acid. This amino acid is very interesting because it can control the movement of ions, form an osmolyte, adjust the opening of stomata, and have defense effects on plants [19]. The use of tryptophan as a precursor of plant growth bioregulators is an approach that could be utilized to increase plant growth and productivity, yields, chlorophyll, and protein contents of sunflower [20].

 Banana-based industries produce enormous quantities of peels as a byproduct waste, which form 40% of the weight of fresh bananas [21]. Currently, these peels are not utilized for any other purpose and are generally thrown as solid waste at great cost, which can contribute to actual environmental problems. The probable use of banana peel relies on its chemical structure. Ansari et al. [22] stated that banana peel extract has high contents of biostimulant components, especially protein, carbohydrates, vitamin A as beta carotene, vitamin C, macro and micronutrients, phenolic compounds, fat, and fibers. The main phenolic substances in banana peels are catecholamines, flavonols, hydroxycinnamic acids, and flavan-3-ols. [23]. It is a good source of a mixture of amino acids, particularly tryptophan amino acids, particularly tryptophan [24]. According to these findings, extracts from banana peel contain similar growth-promoting substances that may contribute to the mechanism of growth initiation in plant species through foliar or soil applications [25]. However, to our knowledge, there is a gap in the literature specifically addressing banana peel extract in this context, which our study aims to fill.

The combination of TD + PSF fungi with potassium sulfate, tryptophan, and banana peel extract was designed based on complementary mechanistic roles to enhance wheat growth and nutrient uptake. TD + PSF fungi serve as bioinoculants that promote nutrient solubilization and plant growth through microbial activity and secondary metabolites. Potassium sulfate provides essential mineral nutrition critical for enzymatic functions and osmotic regulation, while tryptophan acts as a precursor for the plant hormone indole-3-acetic acid (IAA), enhancing root development and nutrient assimilation. Banana peel extract contributes additional bioactive compounds and micronutrients that may stimulate plant physiological responses and offer sustainable waste recycling benefits. The specific doses were selected based on previous studies demonstrating effective concentrations for each factor to optimize their synergistic interactions. This integrated approach goes beyond additive screening by targeting multiple physiological and biochemical pathways simultaneously, aiming to achieve a synergistic enhancement of wheat productivity and nutritional quality under challenging sandy soil conditions.

The research goal is to study the effect of co-inoculation of wheat grains with microbial biostimulant *Trichoderma harzianum* and several strains of *Aspergillus niger.* as phosphate-solubilizing fungi, and/or foliar treatment with K_2_SO_4_ and tryptophan as a non-microbial biostimulant. Moreover, the exploitation of wastes after various manufacturing procedures, like fruit peels (e.g., banana), as supplementation or substitutes (at least partially) of mineral fertilizers, amino acids, and phenolic compounds to increase growth, yield quantity, and quality of wheat plants grown in reclaimed sandy soil.

## Materials and methods

The grains of the wheat cultivar (Sakha-94) were obtained from the Agricultural Research Centre in Egypt. Potassium sulfate and tryptophan were obtained from Sigma–Aldrich Company.

### Experiment site

Field trials were sown in two winter seasons of 2020/2021 and 2021/2022 at the research and production station of the National Research Centre, Nubaria region, Egypt (30_86′67″ N 31_16′67″ E), with a mean altitude of 21 m above sea level. The zone of the soil farm is assumed to be arid or semi-arid. At night, temperatures ranged from 8.83 °C to 15.56 °C and from 7.47 °C to 17.44 °C, while daytime temperatures ranged from 16.34 °C to 28.03 °C and from 19.27 °C to 29.84 °C in the two seasons, respectively. At night, relative humidity ranged from 35.74 to 57.42%, and from 32.3 to 53.30% while daytime relative humidity recorded from 85.97 to 91.62% and from 83.65 to 90.79% in the two successive winters (2020/2021 and 2021/2022), respectively.

According to Chapman & Pratt [26], the mechanical and chemical analyses are described in Tables 1 and 2.

**Table 1:** Mechanical analysis of the experimental soil sites. Analysis of the physical properties of soil

Winter season	Depth (cm)	Sand %	Silt%	Clay%
2020/2021	00–30	85.2	10.4	4.4
	30–60	81.1	13.5	5.2
2021/2022	00–30	81.2	13.3	5.3
	30–60	76.6	16.6	6.8

**Table 2:** Chemical analysis of the experimental soil sites**.** Soil chemical and nutritional properties of the experimental site

Seasons	Depth (cm)	pH	Electrical conductivity (ds/m)	**Saturation Percentage**	Anions (milliequivalents/liter)				Cations (milliequivalents/liter)				CaCo_3%_	Organic Mater %
					CO_3_^−−^	CO_3_-	Cl	SO_4_^−−^	Ca^++^	Mg^++^	Na^+^	K^+^		
2020/2021	00–30	7.84	1.17	32	–	0.50	8.40	1.11	1.80	0.90	7.1	0.20	1.00	0.40
	30–60	7.89	1.79	27	–	0.60	8.00	1.40	2.10	1.50	6.2	0.20	6.00	0.07
2021/2022	00–30	7.95	1.59	23	–	0.32	12.70	1.98	4.00	1.80	9.0	0.20	1.90	0.38
	30–60	7.85	1.81	25	–	0.45	15.40	2.15	5.60	2.00	10.2	0.20	1.30	0.32

### Microorganisms used as inoculum

A consortium of *Trichoderma harzianum* and several strains of *Aspergillus niger* was used as a plant growth-promoting agent during this study. *T. harzianum* is a beneficial fungus well known as a biocontrol agent, tremendously utilized in the management of fungal diseases as they have mycoparasitic properties. Several strains of *A. niger* strains (only identified at the morphological level, These strains formed a great clear zone (halo zone) around their growth when grown on a solid Pikovskaya`s agar (PVK) medium; likewise, they caused a high decline in pH values (from 6.8 ± 0.5 to 2.5 ± 0.5) when they had grown in a liquid PVK medium (data not shown). These characteristics account for *A. niger* strains' capacity to solubilize insoluble forms of inorganic phosphorus. This fungus is well known as a phosphate and potassium solubilizer. The two fungi used during this study are well known as plant growth promoting fungus, Also, they play an important role in improving agricultural productivity.

### Inoculum preparation

A set of Potato Dextrose Agar (PDA) plates was inoculated with *T. harzianum* (TD) and another set of PDA plates was inoculated separately with* A. niger s*trains (PSF). Then, the inoculated plates were incubated at 30 °C for one week to maximize spore production of each fungus (*T. harzianum* and *A. niger* strains). The spores of each fungi were separately collected using sterile 0.01% (V/V) Tween 80 with a wire loop. Then, the obtained spores were mixed together with sterilized Peat moss as a carrier to a final spore concentration of approximately 5*10^7^ spores g^−1^ of each of them, then packaged in a plastic sac, and stored at 4 °C until used.

### Inoculating wheat seeds with inoculum

The inoculum was thoroughly mixed with wheat seeds in a large space using an adhesive material (also known as a sticker) to help the inoculants adhere to each seed as follows; a) mix seeds with enough sticker (Arabic gum) to coat all seeds with a film of the adhesive material, b) add inoculant to the moistened seeds and mix carefully to confirm that each seed was uniformaly coated with the inoculant, c) air dry by spreading the coated seeds in the shade zone and, d) sowing the treated seeds as soon as possible to preserve the inoculant in healthy state and to survive it as long as possible.

### Banana peel extract

 Grand Naine (*Musa cavendishii*) was collected from a local market in Cairo, Egypt, and the peel was air-dried and ground into a powder. The resulting powder (500 g) was extracted with 2L of distilled water and left to stand for 48 hours at room temperature. Then, it was extracted using distilled water and allowed to stand at room temperature for 48 hours. The extract was then centrifuged at 4500 rpm for 10 minutes, and the leftover material was reextracted twice with distilled water as previously mentioned. The crude aqueous extract was concentrated using a rotary evaporator under reduced pressure at 45 °C, and the concentrated extracts were then lyophilized and stored at −20°C [27]. Two banana peel extract application rates (500 and 1000 mg L^−1^) were prepared just before their foliar application. The chemical analyses of the ground peel are described in Table 3.

**Table 3:** Nutritional value of banana peel

Parameters		Value
mg/g dry weight	Nitrogen	9.60 ± 0.10
	Phosphorus	3.1 ± 0.03
	Potassium	16.0 ± 0.18
	calcium	4.3 ± 0.04
	Magnesium	1.32 ± 0.02
	Ferric	0.156 ± 0.002
	Zinc	0.046 ± 0.001
	Carbohydrates	68.31 ± 0.79
	Total free amino acids	12.65 ± 0.32
	Protein	7.58 ± 0.10
	Total Phenols	43.1 ± 0.53
	Flavonoids	17.43 ± 0.23
mg/100 g dry weight	β-carotene	1.2 ± 0.01
	Lycopene	0.076 ± 0.0005
%	DPPH %	55.4 ± 0.71
	pH	6.1

### Experimental procedure

Wheat grains were divided into two equal parts; the first part was left without inoculation, while the other part was co-inoculated with a consortium of *T. harzianum* and several strains of *A. niger*. The experiment was split plot design with three replications, in which both non-inoculated and inoculated wheat grains occupy the main plots while the foliar spraying treatments of potassium sulfate at one rate of 200 mgL^−1^(K 200)_,_ tryptophan at one rate of 50 mgL^−1^(Try 50), potassium sulfate at 200 mgL^−1^ + tryptophan at 50 mgL^−1^ (K 200-Try 50) and banana peel extract at two rates 500 and 1000 mgL^−1^) (BPE500 and BPE1000) were allocated at random in sub-plots. On November 20th, wheat (*Triticum aestivum* L.) grains were planted in rows 3.5 m long with a 20-cm spacing between each row. The plot area was 10.5 square meters (3.0 m wide by 3.5 m long). The sowing rate was 140 kg ha^−1^, using the advised agricultural procedures for cultivating wheat grains. The soil was treated with 360 kg ha^−1^ of calcium superphosphate (15.5% P_2_O_5_) before seeding. Following emergence, nitrogen was administered at a rate of 180 kg ha^−1^ in the form of ammonium nitrate 33.5%. The nitrogen was applied in five equal doses, separated by 20 days, before irrigation. Two identical dosages of potassium sulfate (48.52 percent K_2_O) were applied to the area at intervals of 75 days, each weighing 120 kg/ha. The new sprinkler irrigation system, which added water every five days. Tween 80 was added to the treatments to decrease the surface tension and facilitate absorption during foliar spraying. Two foliar applications of various treatments were made to wheat plants 45 and 60 days after planting, while they were in the tillering and elongation stage (about 4 to 5 leaves). Foliar treatments were applied at a solution rate of 475 L ha^−1^. For each treatment, 1.5 L of spray solution was applied to the experimental plot area of 31.5 m^2^ (three replicates).

A spray volume of 2 L was applied to each 42 m^2^ plot, corresponding to an overall application rate of 475 L per hectare (ha). This rate was calculated based on the proportional scaling from plot size to hectare: Since 1 hectare equals 10,000 m^2^.

### Water irrigation requirements

Allen et al. [28] used the Penman–Monteith equation and crop coefficient to determine the irrigation water needs. The sprinkler irrigation system applied an average of 5950 m^3^ ha^−1^ season^−1^ of irrigation water for both trial seasons.

The following formula was used to determine the quantity of irrigation water:

$$\mathrm{I}\mathrm{W}\mathrm{R}=\left[\frac{\mathrm{E}\mathrm{T}0 \times \mathrm{K}\mathrm{c} \times \mathrm{K}\mathrm{r} \times \mathrm{I}}{\mathrm{E}\mathrm{a}}+LR\right]\times 10$$

where:

IWR = Irrigation water requirement m^3^ ha^−1^/irrigation.

ET_0_ = Reference Evaporation—transpiration (mm/day).

Kc = Crop coefficient.

Kr = Reduction factor.

I = Irrigation interval, day.

Ea = Irrigation efficiency, 90%.

LR = Leaching requirement = 10% of the total water amount delivered to the treatment.

### Plant sample

After 75 days of sowing, plant samples were collected to measure growth characteristics as plant height (cm), number of leaves/tiller, fresh & dry weight of tiller and roots (g). Chemical analysis was also conducted for photosynthetic pigments, IAA, and phenolic compounds at 75 days after sowing. Plant height, spike length, number of spikelets/spike, spike weight, 1000-grain weight (g), and grain yield/spike (g) were all measured at harvest (170 days) on random samples of ten girded tillers in each plot. The entire plot area was harvested to measure and calculate the straw yield, biological yield (ton ha^−1^), and grain yield (ton ha^−1^). The chemical analysis of the grains was conducted to determine carbohydrate and protein percentage, lycopene, β-carotene, flavonoids, antioxidant activity, mineral nutrition, and amino acids. At harvest time, water productivity was determined.

### Water productivity

The Water Productivity (WP) was calculated as the ratio of grain yield (kg ha⁻^1^) to the total irrigation water applied (m^3^ ha⁻^1^) during each growing season. Because the experiment was conducted under sprinkler irrigation in Egyptian sandy soils, rainfall during the experimental periods was negligible and therefore was not included in the calculation. According to Howell et al. [29], water productivity was established. WP links the amount of irrigation water and grain production. The following formula was used to calculate WP:

Water productivity = wheat grain yield (kg ha^−1^)/total amount of irrigation water used, m^3^ ha^−1^/season.

### Biochemical analysis

#### Photosynthetic pigments

Total chlorophyll a, b, and carotenoid contents in fresh leaves of wheat plants were estimated using the method of Lichtenthaler and Buschmann [30]**.** The fresh tissue was ground in a mortar and pestle using 80% acetone. The optical density (OD) of the solution was recorded at 662 and 645 nm (for chlorophyll a and b, respectively) and 470 nm (for carotenoids) using a spectrophotometer (Shimadzu UV- 1700, Tokyo, Japan). The concentration of the pigment fractions (chlorophyll a and b and carotenoids) was accounted for as μg/ml using the following equations:

1 $$\mathrm{C}\mathrm{h}\mathrm{l}\mathrm{o}\mathrm{r}\mathrm{o}\mathrm{p}\mathrm{h}\mathrm{y}\mathrm{l}\mathrm{l} \mathrm{a}=\left[\left(10.3\times \mathrm{E}663\right)-\left(0.918\times \mathrm{E}644\right)\right]=\mu \mathrm{g} {\mathrm{ml}}^{-1}$$

2 $$\mathrm{C}\mathrm{h}\mathrm{l}\mathrm{o}\mathrm{r}\mathrm{o}\mathrm{p}\mathrm{h}\mathrm{y}\mathrm{l}\mathrm{l} \mathrm{b}=\left[\left(19.7\times \mathrm{E}644\right)-\left(3.870\times \mathrm{E}663\right)=\mu \mathrm{g} {\mathrm{ml}}^{-1}\right]$$

3 $$\begin{aligned}\mathrm{C}\mathrm{a}\mathrm{r}\mathrm{o}\mathrm{t}\mathrm{e}\mathrm{n}\mathrm{o}\mathrm{i}\mathrm{d}\mathrm{s}&=\left(4.2\times \mathrm{E}452\right)\\&-\left[\left(0.0264\times \mathrm{c}\mathrm{h}\mathrm{l}\mathrm{o}\mathrm{r}\mathrm{o}\mathrm{p}\mathrm{h}\mathrm{y}\mathrm{l}\mathrm{l} \mathrm{a}\right)+\left(0.426\times \mathrm{c}\mathrm{h}\mathrm{l}\mathrm{o}\mathrm{r}\mathrm{o}\mathrm{p}\mathrm{h}\mathrm{y}\mathrm{l}\mathrm{l} \mathrm{b}\right)\right]\\&=\mu \mathrm{g} {\mathrm{ml}}^{-1}\end{aligned}$$

The concentrations of chlorophylls and carotenoids were expressed as mg/g fresh weight (FW) of plant material.

### Indole acetic acid content (IAA)

According to the method described by Gusmiaty et al. [31]. Fresh leaves were weighed out and extracted three times at 0 °C using 85% cold methanol (v/v). The mixed extracts were collected and made up with cold methanol to a known volume. After that, combine 1 mL of the methanolic extract with 4 mL of the PDAB reagent (dissolve 1 g para-dimethylamino benzoic acid in 50 mL HCl, 50 mL of ethanol 95%) and leave for 60 min at 30- 40 °C. A wavelength of 530 nm was used to spectrophotometrically measure the developing colour. The concentration of IAA was quantified using a standard calibration curve generated with IAA standards (0–60 μg mL⁻^1^), according to the following equation: (y = 0.0099x + 0.0021, R^2^ = 0.999).

### The total phenolic

The spectrophotometer was used to measure the phenolic compounds as described by Gonzalez et al. [32]. A known weight of fresh leaves (1 g) was extracted using 85% cold methanol (50 ml v/v) three times at 90 °C. The combined extracts were collected and made up to a known volume with cold methanol. Then one mL of the extract was mixed with 0.5 mL of Folin's reagent, shaken, and allowed to stand for 3 min. Then 2 mL of saturated sodium carbonate (Na2CO3) was added to each tube, followed by distilled water, shaken, and left for 60 min. The absorbance was determined at 750 nm using a spectrophotometer and expressed as mg gallic acid per 100 g^_1^ dry weight. The concentration of phenol was quantified using a standard calibration curve generated with gallic acid standards (0–60 μg mL⁻^1^), according to the following equation: (y = 0.0203x—0.0028, R^2^ = 0.9996).

### Flavonoid contents

Flavonoid content was determined by the aluminum chloride colorimetric method [33]**.** In a dry, clean test tube, add the following: 50 μl of crude extract, 4 ml of distilled water, and 0.3 ml of 5% NaNO2 solution. Allow to stand for 6 min. After 5 min of incubation, add 0.3 mL of 10% AlCl3 solution. Then, 2 mL of 1 mol L^−1^ NaOH solution was added, and the volume was completed to 10 mL with distilled water. Absorbance was measured at 510 nm using a spectrophotometer and expressed as mg catechol per 100 g^_1^ dry weight. The concentration of Flavonoid was quantified using a standard calibration curve generated with catechol standards (0–70 μg mL⁻^1^), according to the following equation: (y = 0.01x—0.0011, R^2^ = 0.999).

### Lycopene and β-carotene analysis

The procedure provided by Nagata and Yamashita [34] for determining lycopene and -carotene. In a dry, clean test tube, put 1 g of ground wheat grains and then add 10 mL of the mixed solvents (acetone and hexane, in the ratio 4:6). The mixture was mixed until homogenized evenly, then the solution was measured by spectrophotometer at wavelengths 453, 505, 663, and 645 nm for the determination of the lycopene and β-carotene contents in each sample. Equations 1 and 2 were used to calculate the lycopene and β-Carotene contents in milligrams per 100 mL of mixed solvent. The measurement results are calculated by the following equations:

$$\begin{aligned}1-\mathrm{l}\mathrm{y}\mathrm{c}\mathrm{o}\mathrm{p}\mathrm{e}\mathrm{n}\mathrm{e}\left(\mathrm{m}\mathrm{g}/100 \mathrm{m}\mathrm{l}\right)&=0.0458 {\mathrm{A}}_{688}+{0.204 \mathrm{A}}_{645}\\&+0.372 {\mathrm{A}}_{505-}0.0806 {\mathrm{A}}_{453}\end{aligned}$$

$$\begin{aligned}2-\beta -\mathrm{C}\mathrm{a}\mathrm{r}\mathrm{o}\mathrm{t}\mathrm{e}\mathrm{n}\mathrm{e}\left(\mathrm{m}\mathrm{g}/100 \mathrm{m}\mathrm{l}\right)&=0.216 {\mathrm{A}}_{663}-1.22 {\mathrm{A}}_{645}\\&-0.304 {\mathrm{A}}_{505-}0.452 {\mathrm{A}}_{453}\end{aligned}$$

### DPPH radical scavenging activity (DPPH%)

The antioxidant activity of samples was determined by the 2, 2'-Diphenyl-1- picrylhydrazyl (DPPH) radical scavenging activity according to the colorimetric method of Liyana-Pathiranan and Shahidi [35]. One hundred microliters of sample extract were added to 4 mL of prepared DPPH solution (0.1 mM DPPH in methanol solution and 450 μl of 50 mM Tris–HCl buffer (p) H 7.4). Then the mixtures were shaken and placed in the dark at room temperature for 30 min. Absorbance of the final solutions was recorded at 517 nm using a spectrophotometer. The percentage inhibition of the DPPH radical by the samples was calculated according to the formula

$$\begin{aligned}\mathrm{I}\mathrm{n}\mathrm{h}\mathrm{i}\mathrm{b}\mathrm{i}\mathrm{t}\mathrm{i}\mathrm{o}\mathrm{n}\mathrm{\%}&=\left(\mathrm{a}\mathrm{b}\mathrm{s}\mathrm{o}\mathrm{r}\mathrm{b}\mathrm{a}\mathrm{n}\mathrm{c}\mathrm{e} \mathrm{o}\mathrm{f} \mathrm{b}\mathrm{l}\mathrm{a}\mathrm{n}\mathrm{k}-\mathrm{A}\mathrm{b}\mathrm{s}\mathrm{o}\mathrm{r}\mathrm{b}\mathrm{a}\mathrm{n}\mathrm{c}\mathrm{e} \mathrm{o}\mathrm{f} \mathrm{s}\mathrm{a}\mathrm{m}\mathrm{p}\mathrm{l}\mathrm{e}/\mathrm{a}\mathrm{b}\mathrm{s}\mathrm{o}\mathrm{r}\mathrm{b}\mathrm{a}\mathrm{n}\mathrm{c}\mathrm{e} \mathrm{o}\mathrm{f} \mathrm{b}\mathrm{l}\mathrm{a}\mathrm{n}\mathrm{k}\right)\\&\times 100\end{aligned}$$

### Total carbohydrates

The total amount of carbohydrates was calculated in accordance with [36]. A measured weight of dry tissue was introduced into a test tube, to which 10 ml of 1 N sulfuric acid was added. After sealing the tube, it was kept in an oven at 100 °C overnight. The resultant mixture was filtered into a 100 mL measuring flask, and distilled water was added to reach the desired volume. Colorimetry was utilized for determining total sugars. This involved combining one milliliter of sugar solution with an equal volume of 5% aqueous phenol solution and five milliliters of concentrated sulfuric acid. After shaking the tubes for ten minutes, they were immersed in a water bath at 23–30°C for 20 min. The optical density of the resultant color was measured at 490 nm using a spectrophotometer. The concentration of Total carbohydrates was quantified using a standard calibration curve generated with glucose standards (0–60 μg mL⁻^1^), according to the following equation: (y = 0.0048x + 0.005, R2 = 0.9946).

### Determination of protein

Total nitrogen was determined using the micro-Kjeldahl method, as described by AOAC [37]. Total protein content was calculated by multiplying the nitrogen percent by 5.7.

### Mineral contents

Macro- and micro-nutrient concentrations of nitrogen (N), phosphorus (P), potassium (K), calcium (Ca), magnesium (Mg), iron (Fe), zinc (Zn), and manganese (Mn) were determined in wheat grain and banana peel. Fresh samples of both grains and banana peel were dried at 70 C^0^ to constant moisture, and dried ground (1 g) was digested in a mixture of boiling perchloric acid and hydrogen peroxide for 8 h. When the fumes were white and the solution was completely clear, it was cooled to room temperature and brought to a final volume of 10 mL with deionized water. Total N was determined using the micro-Kjeldahl method as described in [37]. P was determined colorimetrically using stannous chloride-ammonium molybdate reagent, after its extraction by sodium bicarbonate. Phosphorus was determined using a Spekol spectrophotometer (VEB Carl Zeiss; Jena, Germany). K was determined using a flame photometer (ELE Flame Photometer, Leighton Buzzard, UK). Ca, Mg, Fe, Zn, and Mn concentrations were determined by atomic absorption spectrophotometry [38].

### Estimation of amino acids

According to de Sousa Fontes et al., 2023 [39], Amino acid compositions of wheat grain were hydrolyzed according to the Catalog of amino acid analyzer (1999) as follows: 50 mg of dry powder of wheat shoot was put into a hydrolysis tube that contained 10 ml of HCL (6 N). The tube was closed (by melting the glass with a suitable gas burner). The tube was put in an oven at 110ºC for 72 h and then cooled down in an ice bath. The solution was centrifuged (4000 rpm for 5 min), and the supernatant was collected. The filtrate was transferred into a chromatographic vial fitted with a 200 µL insert for HPLC–UV determination. The analytical determination was conducted on a liquid chromatography Agilent 1260 Infinity LC System (Agilent, Santa Clara – USA), equipped with a quaternary pump (model G1311C), vacuum degasser, automatic sampler (model G1329B), thermostatic column compartment (model G1316A), and diode array detector-DAD (model G1315D). The column used was the Eclipse Plus RP-C18 (100 × 4.6 mm, 3.5 μm) protected by an RP-C18 guard column (12.6 × 4.6 mm, 5 μm), both manufactured by Zorbax (Santa Clara, USA).

### Statistical analysis

A split-plot design was used for the experiment, with three replications for each of the two growth seasons. The homogeneity of error variances between seasons was confirmed using Bartlett's test before the combined analysis. Season was then taken into consideration as an additional source of variation in a combined analysis of variance (ANOVA). The data from both seasons were combined for the final analysis since the Season × Treatment interaction was not significant (*P* > 0.05) for the assessed variables. MSTAT-C statistical software version 14 was used for analysis of variance [40], and Duncan's Multiple Range Test (DMRT) at the 5% probability level [41] was used to distinguish treatment means.

## Results

### Morphological parameters:

The effect of the treatment with a consortium of *T. harzianum* (TD) and several strains of *A. niger* (TD + PSF) and foliar application of K_2_SO_4_ (200 mg L^−1^), tryptophan (Try) (50 mg L^−1^), and K_2_SO_4_ (200 mg L^−1^) + Try (50 mg L^−1^), and two concentrations of banana peel extract (BPE) (500 and 1000 mg L^−1^) on growth parameters of wheat plants are represented in Table 4. Data showed that all treatments led to a significant increase in growth criteria (plant height, number of leaves/tillers, tiller fresh & dry weight, root fresh & dry weight) alone or combined with TD + PSE treatment, compared with the control. Our results showed that TD + PSE significantly increased the studied growth parameters except plant height in the plant treated with K_2_SO_4_ + Try, and the leaves number/tiller in BPE at 1000 mg L^−1^ induced a non-significant change compared with the corresponding uninoculated untreated plants. Since the highest values of plant height and leaves number/tiller were associated with foliar application of Try (50 mg L^−1^), tiller fresh & dry weight were recorded when co-inoculated with BPE(1000 mg L^−1^), and root fresh & dry weight with BPE (500 mg L^−1^).

**Table 4:** Effect of inoculation of wheat grains with a consortium (TD + PSF) and foliar application of potassium sulfate (K), tryptophan (Try), potassium sulfate + tryptophan (K + Try), and banana peel extract (BPE)and their combination on growth parameters at 75 days from sowing of wheat plants grown in sandy soil (combined analysis of two seasons)

Treatment	Plant height (cm)		Leaves No/tiller		Fresh weight of tiller (g)		Dry weight of tiller (g)		Fresh weight of root (g)		Dry weight of root (g)	
	-Ve	+ Ve	-Ve	+ Ve	-Ve	+ Ve	-Ve	+ Ve	-Ve	+ Ve	-Ve	+ Ve
Control (0.0)	25.0k	32.9i	4.3g	5.2f	1.09l	1.90k	0.213k	0.441j	0.533j	0.609i	0.206j	0.228i
K_2_SO_4_ 200 mg L^−1^	27.1j	36.8h	5.4ef	5.8c	2.35i	2.43h	0.504i	0.615g	0.628f	0.992g	0.464d	0.532b
Try 50 mg L^−1^	38.9g	48.2 a	6.4ab	6. 6a	2.62g	3.17d	0.525h	0.769c	1.043e	1.062d	0.400f	0.488c
K_2_SO_4_200 mgL^−1^ + Try 50 mgL^−1^	40.1f	40.0f	5.7cd	6.4ab	2.29j	3.78b	0.640f	0.782b	1.040ef	1.038ef	0.423e	0.466d
BPE 500 mg L^−1^	41.1e	43.5c	5.5de	6.2b	2.75f	3.47c	0.642f	0.711d	1.038f	1.289a	0.344h	0.597a
BPE 1000 mg L^−1^	42.4d	45.8b	6.3b	6.3b	2.98e	4.41a	0.699e	0.818a	1.140c	1.265b	0.365g	0.532b

Data are means ± standard error (*n* = 3)

a, b, c,… Means with different letters in the same column are significantly different at (*P* < 0.05)

–ve means absent of TD + PSF,** + ve** means present of TD + PSF

### Photosynthetic pigments

**Fig. 1:**
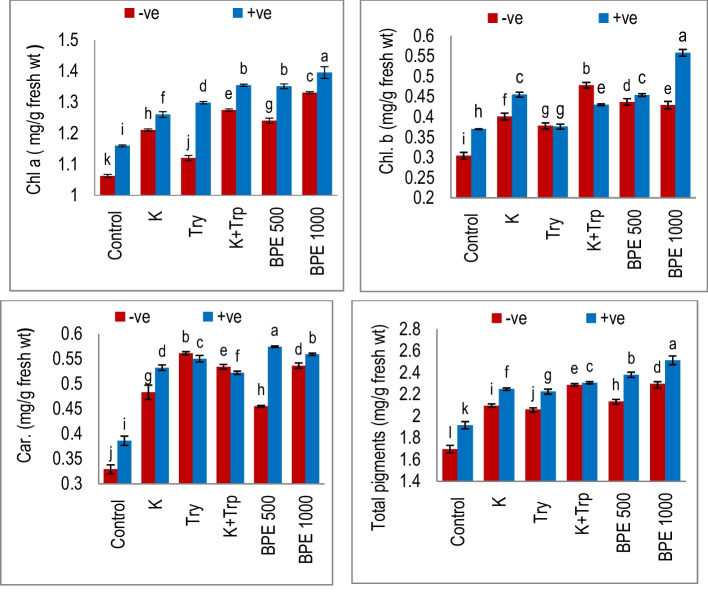
Impact of inoculation of wheat grains with a consortium (TD + PSF) & foliar application of potassium sulfate 200 mgL^−1^(K), tryptophan 50 mgL^−1^ (Try), potassium sulfate + tryptophan (K + Try), and banana peel extract (BPE 500 & 1000 mgL.^−1^) and their combination on photosynthetic pigments as mg/g fresh weight at 75 days from sowing of wheat plant grown in sandy soil. (-Ve without (TD + PSF), + Ve with (TD + PSF). Data are means ± standard error of three replicates (*n* = 3). a, b, c,…. Columns with different letters are significantly different (*P* < 0.05)

According to the findings given (Fig. 1), treatment with (TD + PSF) caused a significant (*P* < 0.05) increase in photosynthetic pigments (chlorophyll a (Chl a), chlorophyll b (Chl b), carotenoids (Car), and total pigments (TP) of wheat leaves. Meanwhile, foliar spraying with K, K + Try, and BPE (500 and 1000) significantly (*P* < 0.05) improved these photosynthetic pigments in contrast with the corresponding untreated plants. The maximum values of Chl a, Chl b, Car, and TP were recorded when foliar BPE(1000 mg L^−1^), K + Try, Try, and BPE (1000 mg L^−1)^ were applied, respectively. The addition of co-inoculation with (TD and PSF) and foliar spraying of different treatments induced progressive increases in all photosynthetic pigments compared to corresponding un-inoculated plants. The maximum values of Chl a (31.4%), Chl b (83.6%), and TP (48.2%) were recorded when foliar BPE(1000 mg L^−1^) application treatments were associated with co-inoculation treatment. The highest value of Car (74.5%) was obtained with the interaction between foliar BPE (500 mg L^−1^) application and co-inoculation (TD + PSF).

### IAA and phenolic contents

**Fig. 2:**
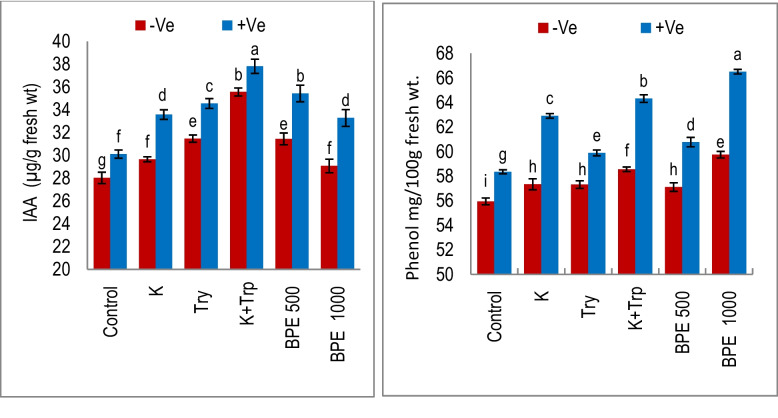
Effect of inoculation of wheat grains with a consortium (TD + PSF) and foliar application of potassium sulfate (K), tryptophan (Try), potassium sulfate + tryptophan (K + Try), and banana peel extract (BPE) and their combination on IAA and phenolic compounds (mg 100 g.^−1^fresh weight) of wheat plants. **(-**Ve without (TD + PSF), + Ve with (TD + PSF). Data are means ± standard error of three replicates (*n* = 3). a, b, c,…. Columns with different letters are significantly different (*P* < 0.05)

Co-inoculation (TD + PSF) treatment and foliar application treatments (K, Try, K + Try, and BPE 500 & 1000 mg L^−1^) led to a significant (*P* < 0.05) increase in IAA compared with the untreated treatment, as shown in Fig. 2. Our results showed that co-inoculation with (TD and PSF) significantly increased the IAA compared with the corresponding uninoculated plants. The most pronounced increase was observed in response to K + Try and TD + PSF by 34.9%.

Treatment with TD + PSF and foliar application treatments (K, Try, K + Try, and BPE 500&1000 mg L^−1^) induced a significant (P < 0.05) increase in phenolic contents related to the untreated treatment, as shown in Fig. 2. The co-inoculation with (TD and PSF) significantly increased the phenolic content compared with the corresponding uninoculated plants. The highest phenolic content value (18.9%) was obtained when the foliar application of BPE1000 mg L^−1^ was associated with co-inoculation treatment.

### Changes in yield, yield components, and water productivity (WP)

Exogenous (TD+PSF) treatment and foliar application treatments (K, Try, K+Try, and BPE 500&1000 mg L^−1^) induced a significant increment in yield and yield components when compared to the untreated treatment (Table 5 a and b). Co-inoculation with (TD and PSF) significantly increased the yield and yield components compared with the corresponding uninoculated plants. BPE1000 mg L^−1^ and the treatment with (TD+PSF) were the most effective treatments in yield and yield components of wheat plants. The percentage increase reached 34.18, 38.87, and 44.07 for straw weight, biological weight, and grain yield, respectively.

**Table 5:** Effect of inoculation of wheat grains with a consortium (TD + PSF) and foliar application of potassium sulfate (K), tryptophan (Try), potassium sulfate + tryptophan (K + Try), and banana peel extract (BPE) and their combination on yield components and water productivity of wheat plants grown in sandy soil (combined analysis of two seasons). **(-**Ve without (TD + PSF), + Ve with (TD + PSF)

Treatment mg L^−1^	Plant height (cm)		Spike length		Spikelet number/spike		Spike weight (g)		1000 grains weight/g	
	-Ve	+ Ve	-Ve	+ Ve	-Ve	+ Ve	-Ve	+ Ve	-Ve	+ Ve
Control (0.0)	70.2i	71.7h	9.6g	9.9g	15.0g	17.0e	2.98i	3.15fgh	40.70l	43.49k
K_2_SO_4_200 mg L^−1^	74.5fg	77.4d	11.5bc	11.6b	18.3ab	18.0bc	3.10h	3.25ef	43.51j	45.17i
Try 50 mg L^−1^	74.8f	77.6d	10.3f	10.9de	17.4d	18.5a	3.31e	3.67c	46.96g	51.32d
K_2_SO_4_ 200 mg L^−1^ + Try50 mg L^−1^	75.2e	80.9b	11.1d	11.8b	17.0e	18.3ab	3.21efg	3.50d	46.73h	51.69c
BPE 500 mg L^−1^	74.3g	79.8c	10.7e	11.2cd	16.1f	18.1bc	3.14gh	3.88b	47.46f	53.13b
BPE 1000 mg L^−1^	77.6d	83.2a	11.6b	12.8a	17.9c	18.5a	3.27e	4.01a	49.36e	54.39a
Table (b)										
Treatment		Straw yield ton/ha		Biological yield ton/ha		Grain yield ton/ha		water productivity (Kg grain yield/m^3^)		
		-Ve	+ Ve	-Ve	+ Ve	-Ve	+ Ve	-Ve	+ Ve	
Control (0.0)		4.704l	5.160k	8.952k	9.744i	4.248l	4.584k	0.714j	0.770i	
K_2_SO_4_ 200 mg L^−1^		5.016j	5.496g	9.648j	10.272h	4.632j	4.776i	0.778i	0.803h	
Try 50 mg L^−1^		5.232i	5.568f	10.248h	10.968f	5.016h	5.400e	0.843g	0.908cd	
K_2_SO_4_ 200 mg L^−1^ + Try50 mg L^−1^		5.352h	6.248b	10.464g	11.960b	5.112g	5.712b	0.859f	0.960b	
BPE 500 mg L^−1^		5.664e	6.192c	11.016e	11.612c	5.352f	5.420d	0.899e	0.908cd	
BPE 1000 mg L^−1^		5.976d	6.312a	11.520d	12.432a	5.544c	6.120a	0.932c	1.029a	

a, b, c,… Means with different letters in the same column are significantly different at (*P* < 0.05).

–ve means absent of TD + PSF,** + ve** means present of TD + PSF

As indicated in Table 5b, most treatments, either alone or in combination, significantly increased water productivity in wheat plants compared to the control. The highest water productivity was observed with the foliar application of BPE at 1000 mg L^−1^, combined with the co-inoculation treatment (44.12%).

### Carbohydrate and protein percentage in the yielded grains

**Fig. 3:**
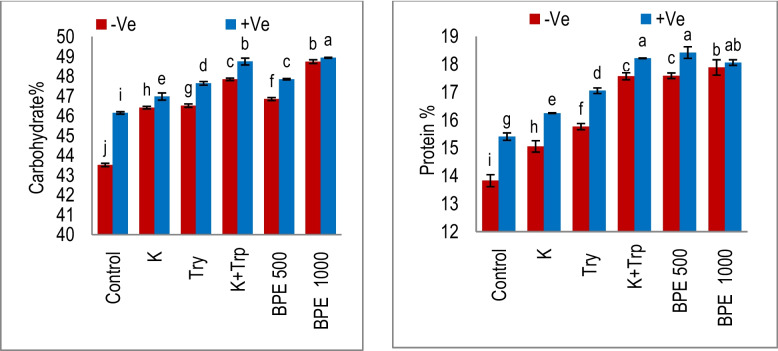
Effect of inoculation of wheat grains with a consortium (TD + PSF) foliar application of potassium sulfate (K), tryptophan (Try), potassium sulfate + tryptophan (K + Try), and banana peel extract (BPE) and their combination on carbohydrates % and protein % of the wheat plant.** (-**Ve without (TD + PSF), + Ve with (TD + PSF). Data are means ± standard error of three replicates (n = 3). a, b, c, Columns with different letters are significantly different at (P < 0.05)

Treatment with TD + PSF and foliar application treatments (K, Try, K + Try, and BPE 500&1000 mg L^−1^) induced a significant increase in carbohydrate and protein percentage in the yielded grain in contrast to the untreated plant Fig. 3. Our results showed that TD and PSF treatments significantly increased the carbohydrate and protein percentages compared with the corresponding uninoculated plants, except that protein % at 1000 mg L-1 showed no significant change. The foliar treatment of BPE at 1000 mg L^−1^ associated with co-inoculation of wheat grain with TD + PSF treatment was the most effective concerning carbohydrate percentage by 12.5% in the yielded grains. While protein and BPE 500 mg L^−1^ + co-inoculation were the most effective treatments for protein percentage by 33.2% in the yielded grains as compared with the control plant.

### Changes in antioxidant compounds (Lycopene, β-carotene, and flavonoids) in the yielded grains

**Fig. 4:**
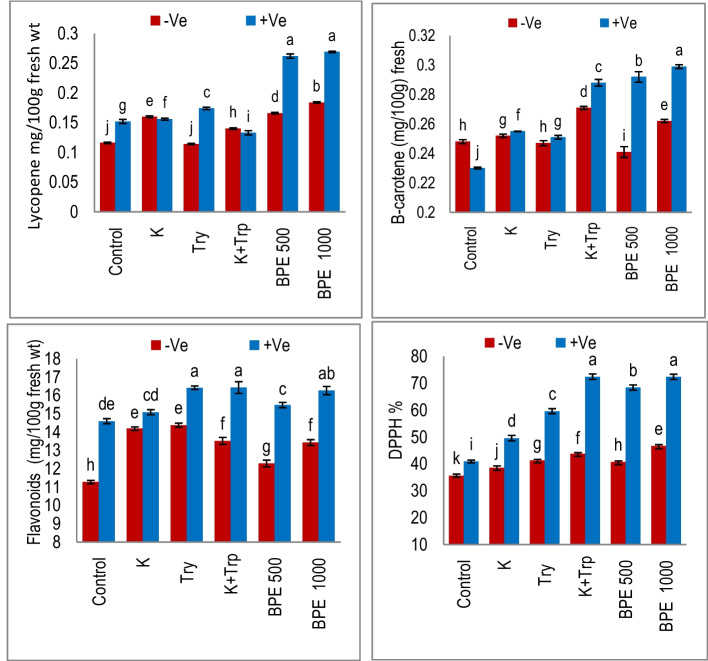
Impact of inoculation of wheat grains with a consortium (TD + PSF) and foliar application of potassium sulfate (K), tryptophan (Try), potassium sulfate + tryptophan (K + Try), and banana peel extract (BPE) and their combination on Lycopene, B-Carotene, Flavonoids, and antioxidant activity % (DPPH) of wheat plant.** (-**ve without (TD + PSF), + ve with (TD + PSF).Data are means ± standard error of three replicates (*n* = 3). a, b, c, Columns with different letters are significantly different at (*P* < 0.05)

A significant increase in each lycopene, β-carotene, and flavonoid content of wheat yielded grains was observed in all treatments, in contrast to the untreated plant, as shown in Figure 4. The most effective treatment was a foliar application of BPE1000 + co-inoculation of wheat grains with (TD+PSF) for lycopene and β-carotene contents of wheat grains. The highest value of flavonoid contents of wheat grains was obtained by Try and K+ Try foliar application associated with co-inoculation of wheat grains with (TD+PSF).

Concerning antioxidant activity, the different treatments significantly increased the total antioxidant activity(DPPH)in contrast to the untreated plant. The maximum value of DPPH% (103.4%) was recorded in the case of foliar application of K+ Try and BPE 1000 mg^−1^ associated with (TD+PSF) co-inoculation of wheat grains treatment, as illustrated in Figure 4.

### Changes in mineral contents in the yielded grains

**Fig. 5:**
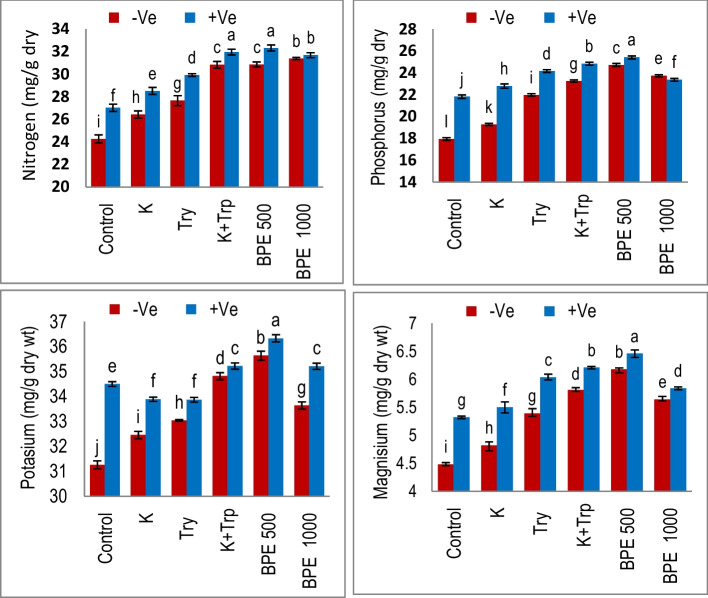
Effect of inoculation of wheat grains with a consortium (TD + PSF) and foliar application of potassium sulfate (K), tryptophan (Try), potassium sulfate + tryptophan (K + Try), and banana peel extract (BPE) and their combination on mineral nutrition (N, P, K, and Mg %) of wheat grains. **(-**Ve without (TD + PSF), + Ve with (TD + PSF).Data are means ± standard error of three replicates (*n* = 3). a, b, c, Columns with different letters are significantly different at (*P* < 0.05)

Figure 5 presents the effects of T. harzianum and A. niger (TD + PSF) addition, foliar application treatments (K₂SO₄, tryptophan, K₂SO₄ + tryptophan, and banana peel extract at 500 and 1000 mg L⁻^1^), and their combinations on the mineral content (N, P, K, and Mg) of wheat grains. All treatments led to significant increases in these minerals compared to the untreated control. Specifically, foliar application of banana peel extract at 500 mg L⁻^1^ (BPE500), whether applied alone or combined with co-inoculation, was generally the most effective treatment in enhancing overall mineral content across the wheat grains. This reflects the broad stimulatory effect of BPE on nutrient accumulation. However, when analyzing each mineral individually, the combined treatment of K₂SO₄ + tryptophan along with TD + PSF yielded the highest percentage increases for nitrogen (33.2%), phosphorus (41.7%), potassium (16.21%), and magnesium (44.20%). These specific increases indicate a strong synergistic effect of this combined treatment on particular nutrient uptakes. A statistically significant difference was observed between foliar treatments alone and those combined with co-inoculation for all analyzed minerals, highlighting the enhanced effect of microbial inoculation combined with foliar nutrition. In summary, while BPE500 consistently improved mineral content broadly, the combination of K₂SO₄ + tryptophan with TD + PSF was particularly effective in maximizing the accumulation of key individual minerals. This distinction clarifies the complementary roles and specific impacts of each treatment, providing a coherent and consistent presentation of the mineral content results.

### Amino acid constituents in grain yield

Data presented in Table 6 show the manner of variation in the amino acid constituents of the grain yield of wheat plants co-inoculated with (TD + PSF) and/or foliar spraying with potassium sulfate, tryptophan, (potassium sulfate + tryptophan), and banana peel extract at 500 &1000 mg L^−1^. Results revealed that proline, glutamic acid, glycine, leucine, isoleucine, and lysine were the highest values among the amino acids (most predominant). Other amino acids were considered minor amino acids. An increase in essential and nonessential amino acid contents was observed in most treatments, particularly those associated with co-inoculation of wheat grains, except for glycine and alanine, compared with the control. Also, co-inoculation of wheat grains with (TD+PSF), alone or in combination with different foliar applications, led to an increase in proline and glutamic acid contents compared with un-inoculated treatments. Our results showed that co-inoculation with (TD and PSF) increased the essential amino acids, such as threonine, valine, methionine, leucine, isoleucine, phenylalanine, Lysine, and methionine, in the treatments with microbial biostimulants of wheat plants, which were higher than those of the non-inoculated plants (Table 6). The foliar application of K+Try followed by BPE 1000 mg L^−1,^ associated with the co-inoculation of wheat grains with TD+PSF, was the most effective treatment in essential amino acid (threonine, valine, methionine, leucine, isoleucine, phenylalanine and histidine) contents. Some amino acid contents were negatively affected, such as cysteine, aspartic acid, and serine, when the co-inoculation of wheat grains treatment was associated with potassium sulphate, tryptophan, and banana peel extract, as compared with the corresponding un-inoculated plant, respectively The ratio of essential to non-essential amino acids in the grain yield was enriched by approximately 12–15% in plants treated with the combination of TD+PSF and other treatments. The highest essential and non-essential AAs were obtained when the foliar application of K +Try was associated with co-inoculation treatment.

**Table 6:** Effect of inoculation of wheat grains with a consortium (TD + PSF) and foliar application of potassium sulfate (K), tryptophan (Try), potassium sulfate + tryptophan (K + Try), and banana peel extract (BPE) and their combination on the amino acid contents (mg/100g dry wt.) of wheat yielded grains. Each value is the ± SE

Treatment	**Control**		**K**_**2**_**SO**_**4**_** 200 mg L**^**−1**^		**Try 50 mg L**^**−1**^		**K**_**2**_**SO**_**4**_** 200 mg L**^**−1**^** + Try 50 mg L**^**−1**^		**500 mgL**^**−1**^** BPE**		**1000 mgL**^**−1**^** BPE**	
	**-Ve**	** + Ve**	**-Ve**	** + Ve**	**-Ve**	** + Ve**	**-Ve**	** + Ve**	**-Ve**	** + Ve**	**-Ve**	** + Ve**
Aspartic acid	**12.53 ± 0.029**	**13.85 ± 0.012**	**11.35 ± 0.023**	**12.85 ± 0.029**	**15.85 ± 0.035**	**7.85 ± 0.015**	**18.85 ± 0.046**	**20.75 ± 0.052**	**14.08 ± 0.011**	**14.92 ± 0.035**	**14.65 ± 0.411**	**15.85 ± 0.025**
Serine	**5.85 ± 0.017**	**8.36 ± 0.015**	**7.65 ± 0.017**	**9.52 ± 0.023**	**8.65 ± 0.017**	**8.68 ± 0.021**	**7.58 ± 0.017**	**9.00 ± 0.023**	**9.48 ± 0.023**	**8.343 ± 0.020**	**9.87 ± 0.241**	**8.68 ± 0.0190**
Glutamic	**39.58 ± 0.10**	**42.35 ± 0.115**	**35.52 ± 0.092**	**51.52 ± 0.132**	**36.52 ± 0.092**	**53.35 ± 0.14**	**36.78 ± 0.098**	**49.63 ± 0.13**	**38.30 ± 0.098**	**46.40 ± 0.127**	**39.85 ± 0.110**	**48.35 ± 0.132**
Proline	**30.50 ± 0.086**	**85.35 ± 0.231**	**31.52 ± 0.081**	**95.85 ± 0.25**	**32.52 ± 0.087**	**100.52 ± 0.26**	**33.52 ± 0.087**	**105.52 ± 0.28**	**39.10 ± 0.101**	**89.10 ± 0.123**	**39.85 ± 0.870**	**93.52 ± 0.242**
Glycine	**90.52 ± 0.24**	**62.52 ± 0.456**	**87.85 ± 0.731**	**60.52 ± 0.156**	**86.85 ± 0.231**	**71.52 ± 0.18**	**86.65 ± 0.23**	**76.96 ± 0.020**	**87.97 ± 0.832**	**69.83 ± 0.662**	**91.52 ± 1.237**	**72.65 ± 0.190**
Cystine	**16.85 ± 0.040**	**23.52 ± 0.058**	**19.85 ± 0.062**	**18.98 ± 0.023**	**18.57 ± 0.046**	**23.58 ± 0.058**	**15.85 ± 0.04**	**26.52 ± 0.063**	**16.19 ± 0.041**	**28.69 ± 0.184**	**16.85 ± 0.041**	**19.85 ± 0.046**
Alanine	**14.52 ± 0.035**	**8.65 ± 0.017**	**16.85 ± 0.040**	**7.68 ± 0.015**	**17.52 ± 0.040**	**8.95 ± 0.023**	**21.52 ± 0.052**	**9.85 ± 0.023**	**22.62 ± 0.058**	**8.41 ± 0.0692**	**23.52 ± 0.0584**	**8.75 ± 0.018**
Tyrosine	**8.75 ± 0.023**	**12.52 ± 0.012**	**9.85 ± 0.231**	**14.58 ± 0.035**	**7.85 ± 0.006**	**16.96 ± 0.023**	**9.65 ± 0.023**	**20.35 ± 0.034**	**10.11 ± 0.024**	**19.08 ± 0.035**	**10.52 ± 0.016**	**19.85 ± 0.062**
Arginine	**21.52 ± 0.052**	**29.85 ± 0.069**	**23.52 ± 0.1577**	**32.52 ± 0.087**	**22.47 ± 0.052**	**34.96 ± 0.087**	**25.68 ± 0.064**	**39.52 ± 0.104**	**22.68 ± 0.061**	**38.87 ± 0.131**	**21.52 ± 0.052**	**40.52 ± 0.131**
Threonine*	**7.85 ± 0.012**	**12.85 ± 0.040**	**10.52 ± 0.021**	**17.85 ± 0.040**	**12.52 ± 0.029**	**17.85 ± 0.04**	**15.75 ± 0.0**	**19. 9 ± 0.012**	**13.31 ± 0.035**	**15.20. ± 043**	**13.85 ± 0.132**	**16.85 ± 0.043**
Valine*	**11.52 ± 0.029**	**15.85 ± 0.041**	**13.52 ± 0.028**	**17.52 ± 0.011**	**10.45 ± 0.023**	**16.85 ± 0.04**	**13.52 ± 0.029**	**20.52 ± 0.052**	**15.02 ± 0.036**	**18.91 ± 0.017**	**13.54 ± 0.035**	**19.68 ± 0.052**
Methionine*	**0.21 ± 0.003**	**1.35 ± 0.012**	**0.35 ± 0.008**	**2.64 ± 0.017**	**0.45 ± 0.003**	**2.75 ± 0.017**	**0.81 ± 0.002**	**3.85 ± 0.017**	**1.36 ± 0.058**	**2.40 ± 0.046**	**1.42 ± 0.006**	**2.5 ± 0.0181**
Leucine*	**23.52 ± 0.058**	**29.85 ± 0.069**	**26.85 ± 0.0635**	**29.85 ± 0.069**	**24.85 ± 0.058**	**32.52 ± 0.087**	**28.35 ± 0.070**	**36.95 ± 0.092**	**28.37 ± 0.071**	**34.14 ± 0.017**	**29.52 ± 0.0.075**	**35.52 ± 0.092**
Isoleucine*	**29.85 ± 0.069**	**32.58 ± 0.087**	**28.85 ± 0.692**	**33.52 ± 0.087**	**30.75 ± 0.081**	**39.85 ± 0.103**	**31.52 ± 0.081**	**42.52 ± 0.11**	**30.29 ± 0.075**	**41.83 ± 0.081**	**31.52 ± 0.081**	**43.52 ± 0.115**
Phenylalanine*	**3.21 ± 0.017**	**6.85 ± 0.025**	**4.08 ± 0.0173**	**8.74 ± 0.023**	**4.22 ± 0.017**	**11.52 ± 0.029**	**2.85 ± 0.017**	**11.52 ± 0.03**	**3.64 ± 0.017**	**12.99 ± 0.116**	**3.75 ± 0.019**	**13.52 ± 0.031**
Histidine*	**11.52 ± 0.029**	**13.52 ± 0.025**	**11.65 ± 0.029**	**15.85 ± 0.040**	**13.52 ± 0.029**	**18.85 ± 0.046**	**13.68 ± 0.035**	**19.85 ± 0.052**	**14.09 ± 0.0122**	**16.10 ± 0.041**	**12.58 ± 0.035**	**16.75 ± 0.063**
Lysine*	**26.85 ± 0.164**	**33.52 ± 0.187**	**26.75 ± 0.0635**	**34.85 ± 0.092**	**29.85 ± 0.069**	**36.52 ± 0.092**	**28.75 ± 0.041**	**28.65 ± 0.070**	**26.97 ± 0.071**	**26.84 ± 0.135**	**30.14 ± 0.076**	**30.85 ± 0.082**
Essential A A*	**114.53 ± 1.306**	**146.37 ± 2.387**	**122.57 ± 1.329**	**160.82 ± 2.122**	**126.61 ± 3.33**	**176.71 ± 3.463**	**135.23 ± 3.351**	**183.55 ± 4.48**	**133.05 ± 2.352**	**168.44 ± 2.441**	**136.32 ± 1.371**	**179.19 ± 2.478**
Non-essential A A	**240.62 ± 3.65**	**286.97 ± 2.733**	**243.96 ± 3.612**	**304.02 ± 4.762**	**246.80 ± 0.62**	**326.37 ± 0.85**	**256.08 ± 3.66**	**358.10 ± 1.93**	**260.53 ± 2.675**	**323.71 ± 3.843**	**268.15 ± 3.712**	**328.02 ± 3.855**
Total amino acids	**355.15 ± 4.95**	**433.34 ± 5.121**	**366.53 ± 0.941**	**464.84 ± 4.180**	**373.41 ± 3.95**	**503.08 ± 4.30**	**391.31 ± 3.02**	**541.65 ± 4.41**	**393.58 ± 3.031**	**492.15 ± 4.292**	**404.47 ± 5.051**	**507.21 ± 4.322**
Essential AA*/non essential AA	**0.476**	**0.510**	**0.502**	**0.529**	**0.513**	**0.541**	**0.528**	**0.513**	**0.512**	**0.520**	**0.508**	**0.546**

AA*: essential amino acid, AA: non-essential amino acid

## Discussion

In sandy soils, plants resist obtaining adequate water and nutrients because of their weak physical properties and chemical compositions, interrupting their growth and overall production. The challenges of inadequate water and nutrient availability for plant growth can be mitigated through treatment with T. harzianum (TD) and various strains of A. niger functioning as phosphate-solubilizing fungi (PSF), combined with foliar applications of K₂SO₄, tryptophan, their combination (K₂SO₄ + tryptophan), and banana peel extract, all of which potentially enhance nutrient management (Table 4). Treating wheat grains with *T. harzianum* (TD) and several strains of *A. niger,* significantly improved growth parameters by enhancing nutrient availability—particularly phosphorus—and promoting nutrient uptake. These findings are consistent with previous research demonstrating Trichoderma’s capacity to colonize plant roots, establish symbiotic relationships with diverse crops, and stimulate growth through the production of phytohormones such as gibberellins (GA₃) and cytokinins (e.g., zeatin), which also contribute to increased plant resilience under environmental stress [42]. Similarly, this aligns with the results of [8], who reported that inoculating flax seeds with phosphate-solubilizing bacteria (PSB) enhanced all measured growth parameters. The observed growth promotion likely results from phytohormone production, including gibberellins and cytokinin-like compounds, which stimulate plant development and improve tolerance to environmental challenges.

Potassium application improves growth by maintaining stomatal function and enhancing CO_2_ fixation, which promotes photoassimilate biosynthesis [43] and root development through improved water uptake, which induces an increment in the root surface exposed to the soil due to enhanced root water uptake; it is also crucial for root growth [44].

L-tryptophan plays a vital role in plant physiological processes by being absorbed and converted into auxins, particularly indole-3-acetic acid (IAA). This conversion elevates endogenous bioregulator levels, which stimulate cell division and expansion, thereby promoting enhanced growth [45]. Our findings are consistent with previous research on quinoa and berseem plants [46, 47], which reported that tryptophan treatment improved multiple physiological functions and overall plant development. Additionally, L-tryptophan may function as an osmolyte, helping regulate stomatal opening and ion transport to maintain osmotic balance. Abdel-Monem et al. [48] further demonstrated the critical role of L- tryptophan in boosting sunflower growth under salt-stress conditions.

The observed enhancement in wheat growth following banana peel extract (BPE) application is consistent with Altaee [49], who reported improved growth parameters and increased chlorophyll content in *Narcissus daffodil* L. This vegetative growth stimulation can be attributed to the essential nutrients present in banana peel, including nitrogen, phosphorus, potassium, and calcium, which play key roles in physiological processes such as photosynthesis [46]. Moreover, the growth-promoting effect of BPE may also stem from its rich content of antioxidant compounds, such as flavonoids, phenolics, and vitamins, which are crucial for plant development and stress mitigation [50, 51].

Different concentrations of BPE provide varying levels of nutrients, bioactive compounds, and growth stimulants to plants. When combined with microbial biostimulants, these substances enhance nutrient uptake, phytohormone production, and stress tolerance in wheat. At optimal BPE concentrations, the extract supplies organic nutrients and bioactive molecules that synergize with microbial biostimulants, leading to significant improvements in wheat growth traits including seed germination, root and shoot length, biomass accumulation, and yield. Conversely, suboptimal BPE concentrations may fail to provide sufficient nutrients or stimulatory compounds, limiting microbial efficacy and plant growth. Excessively high BPE concentrations, on the other hand, may cause phytotoxicity or nutrient imbalances, potentially inhibiting both microbial activity and plant development [52].

All treatments, whether applied individually or with *Trichoderma harzianum* (TD), significantly increased the photosynthetic pigment content in wheat leaves compared to untreated plants, as illustrated in Fig. 1. Trichoderma inoculation promotes deeper root development, which enhances water uptake, thereby boosting photosynthesis and carbohydrate metabolism. These results are consistent with Mishra and Salokhe [53], who demonstrated that treating rice grains with Trichoderma improved photosystem II efficiency and increased transpiration rates in stressed plants. Moreover, Trichoderma stimulates chlorophyll biosynthesis and the production of bioregulators under various stress conditions [54].

Foliar potassium treatment improves photosynthesis and carbohydrate metabolism by improving the leaf's internal CO_2_ concentration and leaf stomatal conductance, thus modifying the stomatal opening and raising the photosynthetic capacity [55, 56]. Additionally, potassium plays a crucial role in the creation of photosynthetic pigment by inhibiting the breakdown of freshly created chlorophyll and the synthesis of δ-aminolevulinic acid (ALA) synthase [57]. Potassium supports glucose metabolism, ribulose-1,5-bisphosphate carboxylase/oxygenase (Rubisco) activity, and light perception [58].

Hussain et al. [20] stated that L-tryptophan influenced the biosynthesis of the chloroplasts of sunflower. Moreover, Xiang et al. [59] illustrated that applying L-tryptophan aids in improving photosynthetic efficacy and adjusting the xanthophyll cycle as a photoprotector. It modulates 15-cis-phytoene synthase (PSY) expression and promotes carotenoid synthesis by acting as an auxin-like hormone.

Likewise, the banana peel contains carotenoids, vitamins A and C, and amino acids, particularly tryptophan, and phenolic compounds, which act as a free radical scavenger that enhances their ability to inhibit the damage induced by reactive oxygen species and increase the antioxidant enzyme activities that promote photosynthesis [60]. Moreover, banana peel is distinguished by necessary elements such as Mg that are needed in the structure of porphyrin, which is participatory in the creation of photosynthetic pigments [47, 61].

All treatments, either separately or combined, led to a marked increase in IAA and total phenols, as shown in Fig. 2. According to earlier research, the phytostimulatory influence of *Trichoderma* implementations has been attributed to a variety of direct and indirect effects on plants, such as the release of compounds with auxin activity (such as indole-3-acetaldehyde, indole-3-carboxaldehyde, and indole-3-ethanol) [62]. These findings corroborate our findings. Furthermore, certain *Trichoderma virens* are known to create auxinic substances that affect plant growth, according to Pelagio-Flores et al. [63]. Zhang et al. [64] showed that *Trichoderma* spp. (TL-6) promoted plant growth via the increment of ACC-deaminase activity and the amount of IAA production in the TL-6 strain, which induces the expression of genes encoding IAA and the amount of IAA production under saline conditions. Gachomo and Kotchoni [65] observed that the root of peanut inoculated with *Trichoderma harzianum* induced an increase in various antioxidant enzymes like peroxidases, chitinases, and some antioxidant compounds such as phenolics and phytoalexins. Moreover, Trichoderma temporarily enhances phenolic glucoside levels in cucumber [66].

Chen et al. [67] presented that the treatment of potassium fertilizer augmented IAA contents in cotton plants. Also, Bulawa et al. [68] stated that potassium treatment at a moderate concentration (14.4 mM) significantly increased the phytochemical content, like total phenols, total antioxidant activities, and total flavonoids, of the T. *divaricate* plant.

Foliar applications of tryptophan or BPE significantly increased the contents of IAA and total phenols. Bakry et al. [46] confirmed this result in quinoa plants. The increase in total phenolic contents led to an increase in IAA contents due to phenolic compounds such as diphenols and polyphenols, which may inhibit IAA oxidase activity and lead to promote auxin formation, which consequently stimulates the plant's development (Table 4) and yield (Table 5a&b). They added that the application of the peels, which depend on their antioxidant components to scavenge ROS, enhance antioxidant activity, and promote photosynthesis, may be responsible for these advantages. Abdel-Monem et al. [48] proposed that tryptophan increases IAA contents of sunflower plants concurrently with the increment in growth rate; the same result was observed in Table 4 and Fig. 2.

Wheat grains treated with microbial biostimulants (*Trichoderma harzianum* and *Aspergillus* led to a significant increase in yield and water productivity of wheat plants compared with the untreated treatment, as shown in Table 5a &b compared to the untreated treatment. The treatments with *Trichoderma* induced improved yield quality and quantity of *Passiflora caerulea* L. plants [69]. The stimulatory results according to the *Trichoderma* under this study may be ascribed to the function of the *Trichoderma* either in stabilizing the chloroplast under regular conditions or in scavenging the ROS produced in plants under stress conditions [56]. They added that *Trichoderma ssp* treatment enhanced the water productivity of wheat (*Triticum aestivum* L.) under salt stress. Likewise, Hashem et al. [54] stated that *T. harzianum* supplies the best potency to organize further intracellular water relations because biomass accumulation results from more water uptake under salt stress [70]. It has been accepted to stimulate crop performance via improving macronutrients, like nitrogen [71] or phosphorus [72], and oligo/micro-nutrients, such as iron [73] or zinc [74], and stimulating crop yield and quality. El-Bassiouny [75] and Bakry et al. [46] have shown that L-tryptophan improved the growth and yield of wheat and quinoa plants, respectively. Tryptophan's positive effects on cell division and natural phytohormones such as auxins may contribute to this increase [76] and improved uptake of nutrients, assimilation, and increased synthesis of proteins [77]. Moreover, L-Tryptophan consists of about 14% nitrogen in its structure, which is liberated during its metabolism in a plant or the rhizosphere and performs a function in improving crop production [20].

Soleimani [78] indicated that potassium is essential for certain plant metabolic processes and that, when available in sufficient amounts, plants can withstand water stress and ultimately produce higher yields. According to Islam et al. [18], potassium treatment increased stomatal regulation, enhanced photosynthetic translocation from source to sink, and increased water uptake, which may result in increased dry matter production, root development, number of grains, and weights under water stress. They added that the increment in yield and its traits was because of the function of potassium in promoting various enzyme activations, protein synthesis, energy transfer, cation–anion balance, and osmoregulation. Egilla et al. [79] stated that K nutrition enhanced plant water productivity in Hibiscus rosa-sinensis under drought conditions. Also, Ul-Allah et al. [80] reported that potassium treatment promoted maize yield characteristics and water productivity under both normal and water stress conditions; however, the influence was more pronounced under water stress conditions than normal conditions.

The enhanced influence of banana peel extracts on yield characteristics might be because of their constituent biostimulant antioxidants, which encourage a preventive function on plant cells. It is appreciated for its biostimulant components, particularly vitamin A as beta carotene, vitamin C, protein, carbohydrates, macro and micronutrients, phenolic compounds, a good source of amino acids, especially tryptophan, and common growth-promoting substances [23, 24]. These components are known to improve growth and encourage cell proliferation, enlargement, and assimilation from source to sink, all of which have an impact on grain yield and its characteristics. Bakry et al. [46] found that the treatment with BPE at 500 mg/l was the utmost efficient treatment for raising quinoa yield. All treatments, including BPE, induced increases in carbohydrate and protein content in wheat grains relative to the control (Fig. 3). Similar enhancements have been observed in other species following tryptophan treatment, such as increased total proteins and carbohydrates in wheat and sunflower [48, 75], and improved growth, yield, and nitrogen uptake in maize [81].

In particular, the use of lycopene and beta-carotene in wheat grain is unusual and requires stronger methodological justification and reference support. Studies have identified lycopene in wheat grains, exhibiting significant variation among different wheat accessions. Furthermore, research has demonstrated the presence of beta-carotene in the pericarp tissue of wheat grains, indicating that carotenoids can accumulate in wheat under certain conditions [82]

All treatments significantly enhanced nitrogen, phosphorus, potassium, and magnesium contents in the wheat grains compared to the control, as shown in Fig. 5. Trichoderma has the ability to solubilize various plant nutrients such as rock phosphate, ferric, copper, magnesium, and metallic zinc, which are often unavailable to plants in certain soils. It achieves this by reducing oxidized metallic ions, thereby increasing their solubility and availability to plants [83, 84]. Additionally, Trichoderma and its secondary metabolites promote the uptake and utilization of key macronutrients like nitrogen and phosphorus [71, 72].

Previous studies support these findings; for example, Abdallah et al. [84] reported that potassium treatment significantly increased protein, nitrogen, phosphorus, and potassium levels in wheat plants. This increase may be attributed to potassium’s role in nutrient uptake and maintaining nutritional balance, which in turn enhances photosynthetic biosynthesis. Similar results were observed by Biswash et al. [85] and Badr et al. [86]. Furthermore, El-Bassiouny [75] found that tryptophan treatment increased potassium, calcium, magnesium, and phosphorus contents. Al-Hallaq et al. [61] also demonstrated that macerated celery seeds with banana peel extract at 5% and 10% improved physiological traits and mineral contents (potassium, nitrogen, and phosphorus) relative to control plants. Moreover, studies have shown that biofertilizers like Trichoderma combined with mineral nutrients can have synergistic effects on plant mineral nutrition and growth [87–89]. For instance, Kumar et al. [90] reported enhanced nutrient uptake and growth in wheat with combined applications of biofertilizers and mineral amendments. Importantly, the Results section revealed a distinct difference between the effects of BPE 500 and the combined treatment of K₂SO₄ + tryptophan with TD + PSF on mineral accumulation. BPE500 elicited a specific pattern of mineral enhancement, whereas the combined K₂SO₄ + tryptophan with TD + PSF treatment showed a synergistic effect that further amplified nutrient uptake. This distinction suggests that these treatments may operate through different physiological mechanisms to enhance mineral nutrition in wheat [90]. Recognizing these differential responses is essential for interpreting the observed variations in mineral content and optimizing treatment strategies. These findings support the detection of lycopene and beta-carotene in wheat grains and provide a basis for further exploration of their roles in wheat nutrition and potential biofortification strategies.

All used treatments, either separately or in combination with (TD + PSF) fungi, increased the levels of amino acid contents (Table 6). These observations were in agreement with those of Abdallah et al. [91] and El-Bassiouny et al. [92], who noticed that the application of arbuscular mycorrhiza fungi led to increments in essential amino acids and total amino acids in *Triticum aestivum* L. plants. Whiteside et al. [93] stated that arbuscular mycorrhiza promotes the uptake of amino acids, like Lys, Asp, Phe, His, Arg, Met, Gly, Glu, and Cys. Ruzicka et al. [94] observed that arbuscular mycorrhiza roots enhance the synthesis of asparagine and glutamic may lead to an increase in glutamine synthetase and asparagine synthetase in the arbuscular mycorrhiza. Sharifi et al. [95] showed that the proline level was higher in soybean plants inoculated with arbuscular mycorrhiza than in non-inoculated plants under salinity stress. Furthermore, the sunflower varieties treated with tryptophan induced higher amino acid accumulation under salt stress [96]. The observed decrease in alanine levels following various treatments may reflect its fundamental role as one of the twenty standard proteinogenic amino acids incorporated into proteins during ribosome-mediated translation [93]. They also mentioned that alanine serves as a key monomer in protein biosynthesis, and its reduction could be attributed to its increased incorporation into newly synthesized proteins, as indicated by the elevated protein levels shown in Fig. 3. This aligns with previous findings that link amino acid pool depletion to enhanced protein synthesis under certain physiological or stress conditions. Despite proline and alanine being components of the amino acid profile of wheat plants, proline is increased; proline is considered one of the main osmolyte compounds which plant used as a form of defence mechanism countering the adverse abiotic conditions, so it is increased in wheat plants grown under sandy soil (Table 6) [97]. Results observed that proline and glutamic acid (most predominant) were the highest values among the amino acids in all treatments. Proline can be easily converted to glutamate, which is involved in the synthesis of other amino acids [98]. While this study provides valuable insights into the effects of biostimulant applications on wheat mineral content and growth, several limitations should be acknowledged. The current research was conducted under controlled or specific field conditions, which may not fully represent the variability encountered in different agroecosystems. Additionally, the study focused primarily on short-term effects, limiting the understanding of how these treatments influence soil health and crop performance over multiple growing seasons. Variability in environmental factors and soil types may also affect the reproducibility of the results. Therefore, to fully understand the long-term effects of biostimulant applications on soil health and crop sustainability, further research is needed. Key areas for future investigation include the sustainability of crop yield and quality, specifically assessing whether the initial yield and nutritional improvements from biostimulants are maintained or enhanced over time without adverse effects. Such studies will help optimize biostimulant use for durable agricultural productivity and environmental health.

The used treatments stand out as a particularly effective and sustainable approach, offering substantial potential to reduce reliance on chemical fertilizers and improve grain quality. While soil health improvements were not directly assessed in this study, future research will investigate the long-term effects of these treatments on soil properties and sustainability. Additionally, the use of banana peel extract provides an eco-friendly and cost-effective method for agricultural waste recycling, supporting sustainable wheat production systems.

This study highlights the significant potential of potassium sulfate, tryptophan, their combination, and banana peel extract—each supplemented with (TD + PSF) fungi—to enhance wheat growth and productivity in sandy soils. These treatments improved physiological performance by increasing photosynthetic pigments, IAA, and phenolic compounds, while also boosting grain nutritional quality through elevated carbohydrate, protein, antioxidant, essential amino acid, and macronutrient contents. Notably, the combination of (TD + PSF) fungi with banana peel extract emerged as a promising sustainable strategy that can reduce dependence on chemical fertilizers, improve grain quality, and support soil health. Additionally, this approach offers an environmentally friendly solution for recycling banana peel waste, contributing to sustainable and cost-effective wheat production systems.

## Conclusion

The present study demonstrated that potassium sulfate, tryptophan, their combined application, and banana peel extract, when supplemented with (TD + PSF) fungi, significantly enhanced wheat growth and productivity under sandy soil conditions. These treatments promoted the accumulation of photosynthetic pigments, indole-3-acetic acid (IAA), and phenolic compounds, indicating improved physiological performance. Furthermore, the treatments enhanced grain nutritional quality by increasing carbohydrate, protein, lycopene, β-carotene, flavonoid, essential amino acid, and macronutrient (N, P, K, and Ca) contents. Among the tested treatments, the integration of (TD + PSF) fungi with banana peel extract at 1000 mg L^−1^ showed considerable potential as a sustainable bio-based approach for improving wheat performance in growth, yield, and grain quality in sandy soils. This strategy may reduce reliance on chemical fertilizers while enhancing grain nutritional value and soil health. Moreover, the utilization of banana peel waste provides an environmentally friendly and cost-effective option for agricultural waste recycling, supporting sustainable wheat production systems.

## Supplementary Information

Supplementary Material 1.

## Data Availability

The corresponding author can provide the datasets used and/or analyzed during the current work upon proper request.
