# Supplementary material for: Combined effect of microbial and non-microbial biostimulants on wheat growth, yield, and grain nutritional quality under sandy soil conditions
Source: BMC Plant Biol. 2026 Aug 13;26:1378. doi: 10.1186/s12870-026-09659-4 (PMC13471340; doi:10.1186/s12870-026-09659-4)
Supplement: Supplementary file 1 [file 12870_2026_9659_MOESM1_ESM.docx]

Handling Editor: Dr. Amira Ibrahim

We appreciate the opportunity allowing us to enrich the manuscript with valuable suggestions. The authors hope that the manuscript, in its new version, meets the Journal's standards.

Kind regards,

Sincerely yours,

El-Bassiouny HMS

**Handling Editor Comments**:

"The corresponding author can provide the datasets used and/or analyzed during the current work upon proper request" (Done).

**Point-by-point response:**

**Reviewer 5**

We sincerely appreciate your careful and thorough evaluation of our manuscript and the constructive feedback provided. Regarding your request to include the standard curves for the measured properties in the Materials and Methods section, we would like to clarify our approach. The authors believe that including the standard curves for measured properties directly in the Materials and Methods section is not preferable, as this section is already crowded with methodological details. Initially, the methods were presented in brief to maintain conciseness. However, in response to reviewer comments, we expanded all methodological details, which significantly lengthened the section. To avoid overcrowding the manuscript while still ensuring transparency and reproducibility, we have provided the detailed explanations and corresponding standard curves in supplementary files (Word).

All the following equations are already fully described in the Materials and Methods section in the manuscript.

**Photosynthetic pigments:**

Total chlorophyll a, b, and carotenoid contents in fresh leaves of wheat plants were calculated by equation:-

Chlorophyll a = [(10.3 × E663) −(0.918 × E644)] = μg /ml (1)

Chlorophyll b = [(19.7 × E644) −(3.870 × E663)] = μg /ml (2)

Carotenoids = (4.2×E452) − [(0.0264×chlorophyll a) +(0.426×chlorophyll b)] = μg/ ml (3).

**Indole acetic acid content (IAA):**

| IAA (µg/ml) | OD |
| --- | --- |
| 0 | 0 |
| 10 | 0.097 |
| 20 | 0.203 |
| 30 | 0.31 |
| 40 | 0.395 |
| 50 | 0.493 |

**The total phenols:**

OD

| Gallic acid µg/ml | OD |
| --- | --- |
| 0 | 0 |
| 10 | 0.195 |
| 20 | 0.41 |
| 30 | 0.593 |
| 40 | 0.815 |
| 50 | 1.01 |

**Flavonoid contents:**

OD

| Catechol µg/ml | OD |
| --- | --- |
| 0 | 0 |
| 10 | 0.105 |
| 20 | 0.19 |
| 30 | 0.295 |
| 40 | 0.41 |
| 60 | 0.6 |

**Lycopene and β-carotene analysis:**

Calculated by following equations:

1- lycopene (mg/100 ml) = 0.0458 A_688_ + 0.204 A_645_ + 0.372 A_505 -_0.0806 A_453_
2- β-Carotene (mg/100 ml) = 0.216 A_663_- 1.22 A_645_ - 0.304 A_505 -_0.452 A_453_

**DPPH radical scavenging activity (DPPH%):**

Calculated according to the formula:-

Inhibition% = (absorbance of blank – Absorbance of sample/absorbance of blank) × 100

**Total carbohydrates:**

| Glucose µg/ml | OD |
| --- | --- |
| 0 | 0 |
| 10 | 0.058 |
| 20 | 0.096 |
| 30 | 0.16 |
| 40 | 0.198 |
| 50 | 0.24 |

**Determination of protein**

Total nitrogen was determined using the micro-Kjeldahl method. Total protein content was calculated by multiplying the nitrogen percent by 5.7.

**Mineral contents:**

Total N was determined using the micro-Kjeldahl method. P was determined colorimetrically using stannous chloride-ammonium molybdate reagent, after its extraction by sodium bicarbonate. Phosphorus was determined using a Spekol spectrophotometer (VEB Carl Zeiss; Jena, Germany). K was determined using a flame photometer (ELE Flame Photometer, Leighton Buzzard, UK). Ca, Mg, Fe, Zn, and Mn concentrations were determined by atomic absorption spectrophotometry.

**Estimation of amino acids**:

The analytical determination was conducted on a liquid chromatography Agilent 1260 Infinity LC System (Agilent, Santa Clara – USA), equipped with a quaternary pump (model G1311C), vacuum degasser, automatic sampler (model G1329B), thermostatic column compartment (model G1316A), and diode array detector-DAD (model G1315D). The column used was the Eclipse Plus RP-C18 (100 × 4.6 mm, 3.5 μm) protected by an RP-C18 guard column (12.6 × 4.6 mm, 5 μm), both manufactured by Zorbax (Santa Clara, USA).
